# A phase II study of axalimogene filolisbac for patients with previously treated, unresectable, persistent/recurrent loco-regional or metastatic anal cancer

**DOI:** 10.18632/oncotarget.27536

**Published:** 2020-04-14

**Authors:** Cathy Eng, Marwan Fakih, Manik Amin, Van Morris, Howard S. Hochster, Patrick M. Boland, Hope Uronis

**Affiliations:** ^1^MD Anderson Cancer Center, Houston, TX, USA; ^2^City of Hope, Duarte, CA, USA; ^3^Siteman Cancer Center, Washington University School of Medicine, St. Louis, MO, USA; ^4^Rutgers Cancer Institute, New Brunswick, NJ, USA; ^5^Roswell Park Cancer Institute, Buffalo, NY, USA; ^6^Duke Cancer Institute, Durham, NC, USA

**Keywords:** anal neoplasms, immunotherapy, *Listeria monocytogenes*, papillomaviridae, phase II clinical trial

## Abstract

Squamous cell carcinoma of the anorectal canal (SCCA) is a rare HPV-related malignancy that is steadily increasing in incidence. A high unmet need exists for patients with persistent loco-regional and metastatic disease. Axalimogene filolisbac (ADXS11-001) is an investigational immunotherapy that stimulates tumor-specific responses against HPV-associated cancers, and has demonstrated benefit in metastatic cervical cancer. We conducted this single-arm, multicenter, phase 2 trial in patients with persistent/recurrent, loco-regional or metastatic SCCA. Patients received ADXS11-001, 1 × 10^9^ colony-forming units intravenously every 3 weeks. A Simon 2-stage design was used to test primary co-endpoints of overall response rate (ORR) and 6-month progression-free survival (PFS) rate. Study would proceed to full enrollment if ORR ≥ 10% or 6-month PFS rate ≥ 20%. Thirty-six patients were treated; 29 patients were evaluable for response. One patient had a prolonged partial response (3.4% ORR). The 6-month PFS rate was 15.5%. Grade 3 adverse event were noted in 10 patients, with the majority being cytokine-release symptoms; one grade 4 adverse event was noted. No grade 5 adverse events occurred. ADXS11-001 was safe and well-tolerated in patients with SCCA. However, this study did not meet either primary endpoint. ADXS11-001 may be more beneficial when administered in combination with other cytotoxic or targeted agents.

## INTRODUCTION

It is estimated that approximately 8600 new cases of anal cancer will be diagnosed in the United States in 2019, and 1160 people will die from the disease [1, 2]. Although accounting for less than 1% of all malignancies, anal cancer has been increasing in incidence by 2.2% per year for the past decade, while annual death rates increased by 2.9% per year from 2005 to 2014 [1]. Primary tumors of the anal canal can be of multiple types, with squamous cell carcinoma of the anal canal (SCCA) comprising the most common histology (85%), followed by adenocarcinoma (10%) [3]. Most patients with localized SCCA (stage I-III) can be cured by chemoradiation [4, 5], however up to 25% of patients develop distant metastases (Stage IV disease) [6, 7]. Patients with Stage IV disease have dismal prognoses, with 5-year survival rates of 15–20% [8].

Limited prospective trials have been completed in patients with metastatic disease due to the rarity of the disease. A single arm phase II trial of docetaxel, cisplatin, and 5-fluoruracil (5-FU) in treatment naïve patients resulted an impressive response rate of 89% [9]. A small randomized phase II trial compared 5-FU plus cisplatin vs. carboplatin plus paclitaxel noting equivalent response rates of 57% vs. 59%, respectively [10].

The most common risk factor for anal cancer is infection with human papilloma virus (HPV), which is found in more than 90% of anal cancer cases [11–14]. The HPV proteins E6 and E7 play central roles in transforming anal squamous epithelium into invasive cancer [15–17], are immunogenic, and may trigger an anti-tumor immune response by recruitment of tumor-infiltrating lymphocytes [18, 19]. Given the immunogenicity associated with these tumors, a recent single-arm phase II study of patients with previously-treated metastatic SCCA examined the response rate and safety of nivolumab, an anti-PD-1 antibody that boosts antitumor immunity by diminishing immune checkpoint activity [20]. Among 37 patients, the overall response rate (ORR) was 24% [95% confidence interval [CI]: 15%–33%]) with an observed prolonged complete response (CR) in one patient longer than 2 years. The median progression-free survival (PFS) was 4.1 months (95% CI 3.0–7.9 months) and 6-month PFS was 38% (95% CI: 24%–60%). A Phase IB study with pembrolizumab found similar benefit in patients with PD-L1 overexpressing unresectable anal cancer, with an ORR of 17% [95% CI: 5%–37%] and median PFS of 3.0 months [95% CI: 1.7–7.3 months]) [21]. Based on these promising results, nivolumab and pembrolizumab are currently recommended as treatment options in previously treated patients with metastatic anal cancer [22]. While these two encouraging studies illustrate the potential benefits of immunotherapy in patients with recurrent or metastatic anal cancer there is still a need for additional therapies to treat this orphan disease.

Axalimogene filolisbac (ADXS11-001) is a new immunotherapeutic agent for HPV-associated locally advanced cancers [23, 24]. This novel agent consists of a live, irreversibly-attenuated (*prfA-*deficient), and nonpathogenic strain of the intracellular bacterium *Listeria monocytogenes* (*Lm*), which has been bioengineered to secrete an antigen-adjuvant fusion protein between Listeriolysin O (LLO) and the HPV-16 E7 oncoprotein [23, 24]. Preclinical studies found that ADXS11-001 stimulated tumor-specific responses against HPV-associated cancers by stimulating the innate, adaptive and humoral arms of the immune system. Furthermore, treatment reduced immune tolerance by neutralizing regulatory T-cells and myeloid-derived suppressor cells within the tumor microenvironment [25–34]. In a single institution pilot study, ADXS11-001 was evaluated in combination with radiation and chemotherapy in locally advanced high-risk anal cancer (T>4 cm and/or lymphadenopathy). All 9 assessable patients had complete clinical responses of the primary tumor at six months. Eight of 9 patients (89%) were progression-free after a median follow-up of 42 months [35]. ADXS11-001 has also shown clinically meaningful responses in patients with recurrent metastatic cervical cancer and is being evaluated in an ongoing phase III clinical trial, NCT02853604 [36, 37]. We report here the first-in-human study to assess tumor response and safety of ADXS11-001 in patients with previously treated advanced SCCA.

## RESULTS

**Figure 1:**
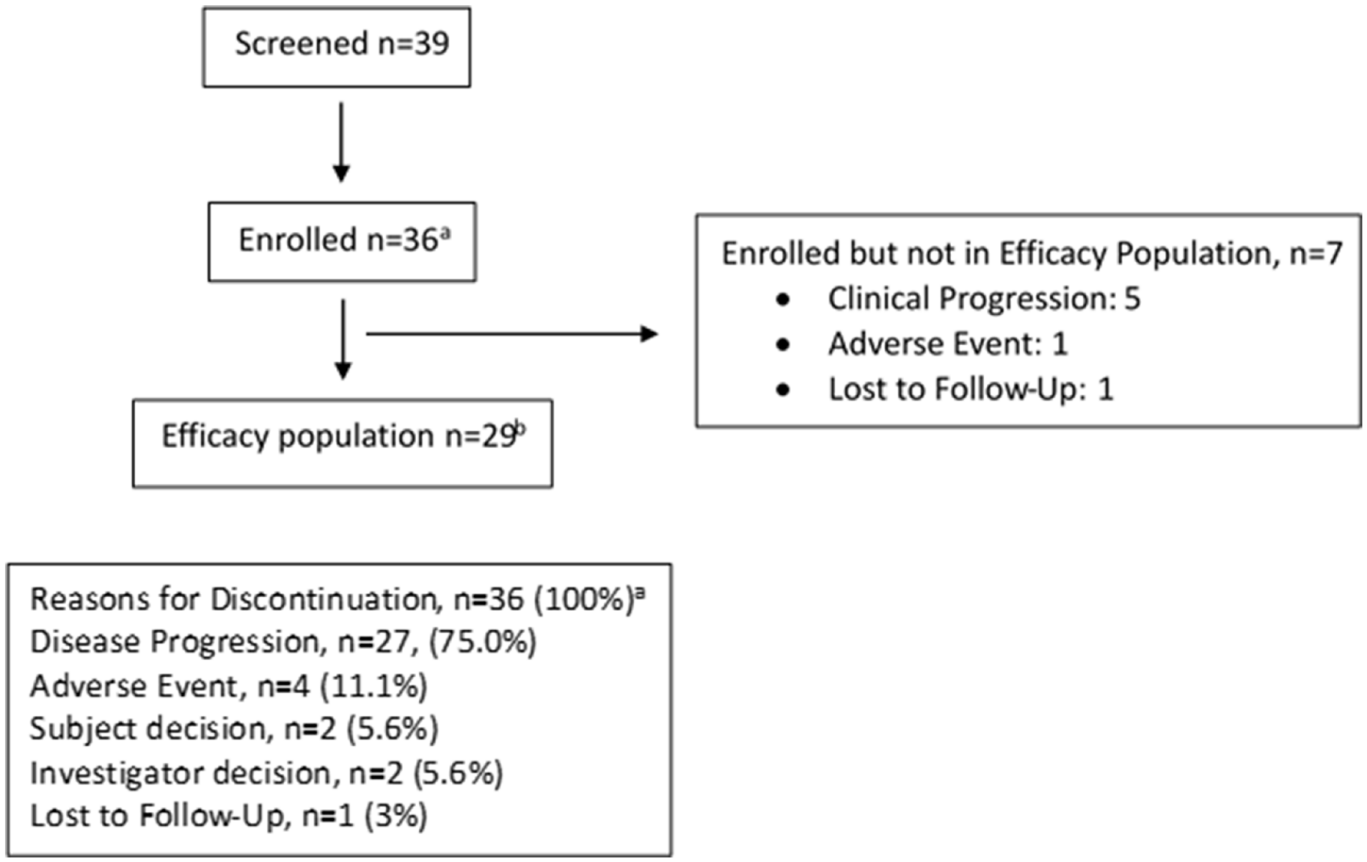
Consort flow diagram. ^a^Safety population: all patients who received at least one dose of ADXS11-001 (*note: all 36 enrolled patients received at least one dose*). ^b^Efficacy population: all patients who received at least one dose of ADXS11-011 and had at least one post-baseline tumor response assessment. Note: 31 patients were planned for Stage 1 but patients consented at the time of the 31st patient were allowed to enroll, leading to 5 additional patients.

Thirty-nine patients were screened from May 2016 to December 2016; three patients were screen failures. Data cut-off date was August 15, 2017. Thirty-six patients were evaluable for the safety analysis; 29 patients were evaluable for response based on diagnostic imaging. Seven patients withdrew from the study prior to receiving cycle 2 of therapy; 5 patients had clinical progression (3 patients within one week of initiating therapy); 1 patient developed a grade 3 treatment related infusion reaction; and 1 patient was lost to follow-up. Baseline demographic data are listed in Table 1. Prior treatment included chemotherapy in 34 patients (94.4%) and chemoradiation in 26 patients (72.2%) (Table 1). The median follow-up time for all patients was 8.1 (range 1.3 to 17.9) months. Patients were treated for a median of 9 weeks (one treatment cycle: range 1–6) and received a median of 3 doses of ADXS-11-001 (range 1–21, interquartile range 3–5). At the time of the data cut-off, 36 patients had discontinued treatment by meeting a protocol-specified criterion: 27 patients (75%) due to disease progression, 2 patients (5.6%) withdrew consent, investigator withdrew 2 patients (5.6%), and 4 patients (11.1%) discontinued due to an adverse event (Figure 1). Treatment remains ongoing for 1 patient as of February 16, 2018.

**Table 1:** Demographics and baseline characteristics

	All Treated (*n* = 36)
Median age, years (range)	60.5 (43, 77)
Female gender, *n* (%)	29 (80.6)
Race, *n* (%)	
Asian	2 (5.6)
Black or African American	1 (2.8)
White	32 (88.9)
American Indian or Alaskan Native	1 (2.8)
ECOG performance status, *n* (%)	
0	25 (69.4)
1	11 (30.6)
Time from initial diagnosis to first dose (*N* = 28)	
Median time, months (range)	29.7 (9, 201)
Tumor stage at entry, *n* (%)	
II	1 (2.8)
IIA	0
IIB	1 (2.8)
III	2 (5.6)
IIIA	0
IIIB	0
IV	29 (80.6)
Other	3 (8.3)
Prior cancer surgery, *n* (%)	
Yes	22 (61.1)
No	14 (38.9)
Prior therapy, *n* (%)	
Any	35 (97.2)
Chemotherapy	34 (94.4)
Immunotherapy	10 (27.8)
Number of prior regimens, *n* (%)	
1	2 (5.6)
2	6 (16.7)
3	7 (19.4)
≥ 4	20 (55.6)

ECOG, Eastern Cooperative Oncology Group.

**Figure 2:**
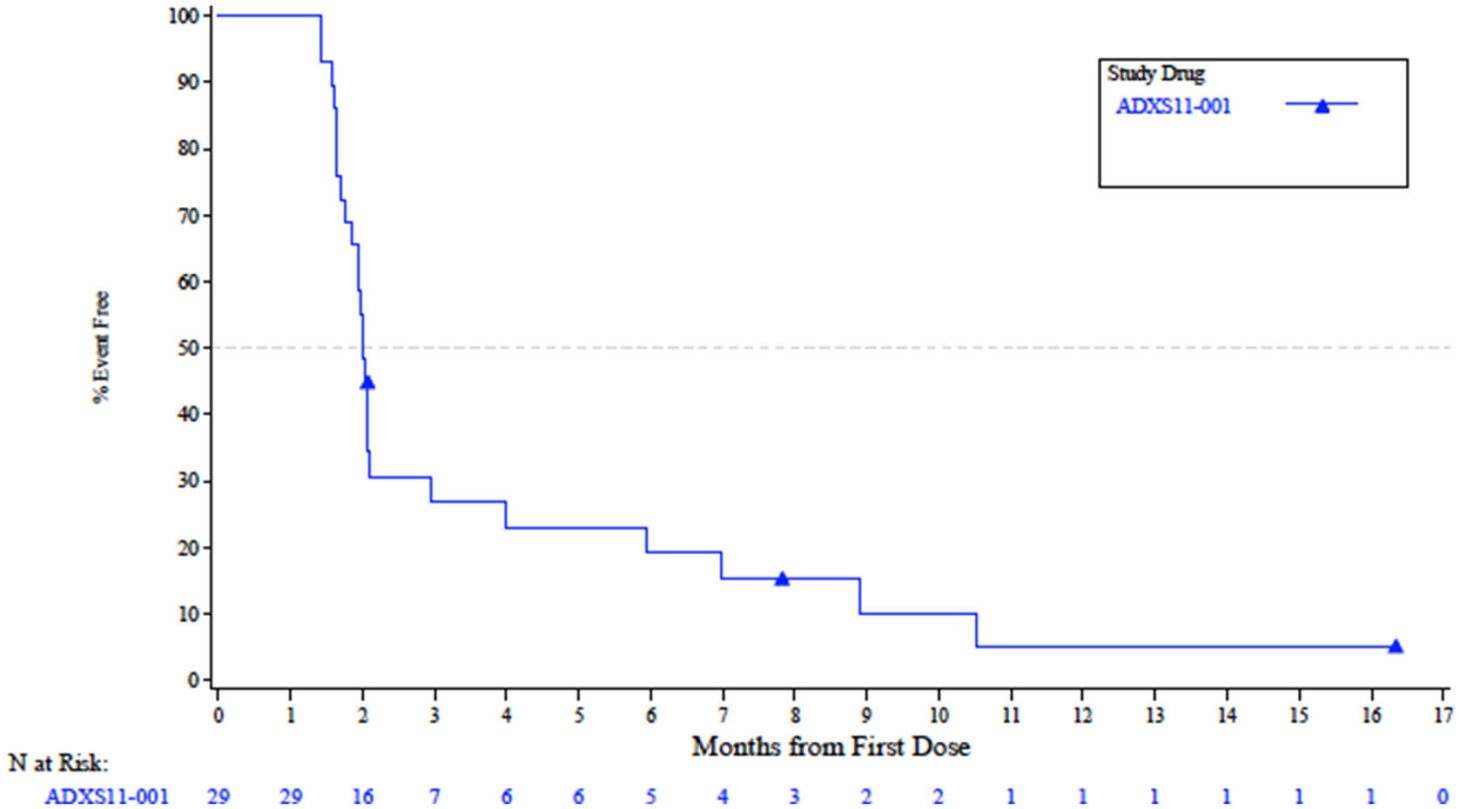
Radiologic progression-free survival in the Efficacy-Evaluable population.

**Figure 3:**
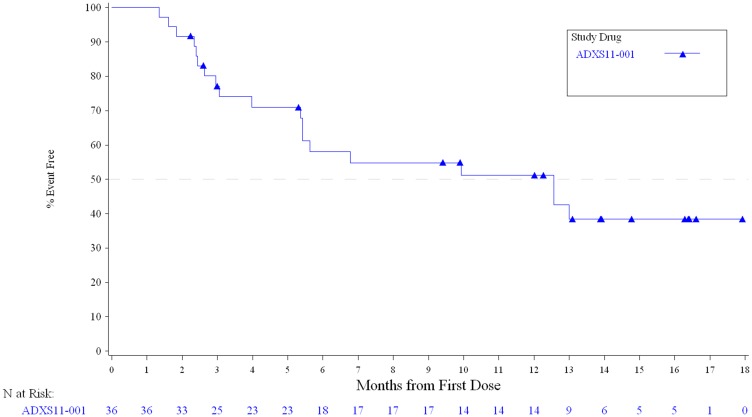
Overall survival in all treated subjects.

In Stage I of the study, 1 patient achieved a partial response (PR; ORR 3.4%; Table 2) with a duration of 6.0 months, and a maximum decrease in size of target lesions by 83%. Six patients had stable disease (SD; 20.7%) with all of these patients meeting the definition of disease control by maintaining response for at least 24 weeks. Twenty patients (55.6%) had disease progression at initial restaging. The 6-month PFS was 15.5% (95% CI 5.7–29.6%). The median PFS (Figure 2) was 2.0 months (95% CI 1.8–2.1 mos.) with a median OS (Figure 3) of 12.6 mos. (95% CI 5.4–Not Estimable).

**Table 2:** Response

	Efficacy-Evaluable Population^a^ (*n* = 29)
Response, *n* (%)^b^	
CR	0 (0)
PR	1 (3.4)
SD	6 (20.7)
PD	20 (69.0)
NE	2 (6.9)
ORR, % (95% CI)^c^	3.4 (0, 17.8)
DCR, % (95% CI) ^d^	24.1 (10.3, 24.5)
Median PFS, months (95% CI)	2.0 (1.8, 2.1)

CI, confidence interval; CR, complete response; DCR, disease control rate; NE, not evaluable; ORR, overall response rate. PD, progressive disease; PFS, progression-free survival; PR, partial response; SD, stable disease.

^a^All enrolled patients who had at least one post-baseline tumor assessment.

^b^Best overall responses were identical with or without response confirmation.

^c^ORR = (CR + PR)/total × 100.

^d^DCR = (CR + PR + SD)/total × 100.

### Toxicities

Of the 36 patients treated with ADXS11-001, the most common treatment-related adverse events occurring in ≥ 25% of patients were chills, pyrexia, nausea, hypotension, vomiting, fatigue, and headache (Table 3). Grade 3 treatment-related adverse events occurred in 10 patients (27.8%); 1 patient each had cytokine release syndrome, ascites, diarrhea, encephalopathy, and acute renal failure; two patients each had an infusion-related reaction, dyspnea, and increased hepatic enzymes; and 4 patients had hypotension. One patient (2.8%) had a Grade 4 treatment-related adverse events of respiratory failure. (Table 3). There were no treatment-related deaths (Table 3). Five patients discontinued the study because of drug-related toxicity. There were no cases of delayed listeria infection during the *Lm* surveillance monitoring period.

**Table 3:** Safety

	Safety Population (*n* = 36)					
	Grade 1	Grade 2	Grade 3	Grade 4	Grade 5	Total
**Treatment-related Adverse Events, *n* (%)**						
Chills	1 (2.8)	21 (58.3)	0	0	0	22 (61.1)
Pyrexia	9 (25.0)	9 (25.0)	0	0	0	18 (50.0)
Nausea	13 (36.1)	4 (11.1)	0	0	0	17 (47.2)
Hypotension	0	12 (33.3)	4 (11.1)	0	0	16 (44.4)
Vomiting	10 (27.8)	3 (8.3)	0	0	0	13 (36.1)
Fatigue	8 (22.2)	4 (11.1)	0	0	0	12 (33.3)
Headache	7 (19.4)	4 (11.1)	0	0	0	11 (30.6)
Infusion-related reaction	0	6 (16.7)	2 (5.6)	0	0	8 (22.2)
Back pain	4 (11.1)	4 (11.1)	0	0	0	8 (22.2)
Diarrhea	2 (5.6)	2 (5.6)	1 (2.8)	0	0	5 (13.9)
Abdominal distension	1 (2.8)	2 (5.6)	0	0	0	3 (8.3)
Cytokine-release syndrome	0	2 (5.6)	1 (2.8)	0	0	3 (8.3)
Decreased appetite	2 (5.6)	1 (2.8)	0	0	0	3 (8.3)
Dizziness	1 (2.8)	2 (5.6)	0	0	0	3 (8.3)
Dyspnea	1 (2.8)	0	2 (5.6)	0	0	3 (8.3)
**Serious treatment-related Adverse Events, *n* (%)**						
Total Adverse Events	0	2 (5.6)	8 (22.2)	1 (2.8)	0	11 (30.6)
Diarrhea	0	1 (2.8)	1 (2.8)	0	0	2 (5.6)
Hypotension	0	0	2 (5.6)	0	0	2 (5.6)
Ascites	0	0	1 (2.8)	0	0	1 (2.8)
Cytokine-release syndrome	0	0	1 (2.8)	0	0	1 (2.8)
Pneumonia	0	1 (2.8)	0	0	0	1 (2.8)
Infusion-related reaction	0	0	1 (2.8)	0	0	1 (2.8)
Encephalopathy	0	0	1 (2.8)	0	0	1 (2.8)
Acute kidney injury	0	0	1 (2.8)	0	0	1 (2.8)
Respiratory failure	0	0	0	1 (2.8)	0	1 (2.8)

WBC, white blood cell. Shown are treatment-related adverse events by worst grade reported in 3 or more patients and serious treatment-related adverse events by worst grade. Data are based on the entire safety population (*n* = 36).

## DISCUSSION

This study was prospectively designed to evaluate ADXS11-001 in patients who had received previous treatment for refractory metastatic SCCA. There are a limited number of treatment options available for this population. Historically, doublet chemotherapy with cisplatin and fluorouracil was recognized as the most common treatment provided for treatment naïve patients. The previously conducted studies of immune checkpoint inhibitors in this population demonstrated findings which are encouraging with respect to providing meaningful clinical benefit for these patients [40]. However, the need for novel treatments still remains. 

Although our multicenter phase II study, did not fulfill the primary endpoint of greater than 20% PFS, there are advantages to this clinical analysis. This is the first, multicenter trial of a novel bioengineered vaccine that we are aware of specific to HPV16/E7. Furthermore, a durable PR was achieved in a patient who had previously failed immune checkpoint inhibitor inhibition. In addition, ADXS11-001 was well-tolerated with a safety profile consistent with what has been reported in other HPV-associated malignancies [24]. The median OS, measured from the time of the first treatment, was 12.6 months. This result was comparable to that seen in the nivolumab study, with a median OS of 11.5 months (95% CI 7.1–not estimable) [40]. It is likely that ADXS11-001 would likely work better in combination with other treatments, such as immune checkpoint blockade agents given their promising single agent results in this patient population [20, 21], but future clinical studies are needed for confirmation.

There were several limitations in this study. This was a small single arm phase II study of which 5 patients had very early evidence of clinical progression and were unevaluable by diagnostic imaging. Second, due to financial constraints tissue and blood correlatives were not collected, which did not allow characterization of ADXS11-001’s ability to activate the innate adaptive CD4-positive and CD8-positive T cells within the tumor, as well as look at an association between treatment responses for ‘responders’ and ‘non-responders’. Profiling and quantifying immune-related gene expression in their peripheral blood mononuclear cells (PBMCs) before and after ADXS11-001 treatment would have been helpful to determine whether any gene expression profiles were associated with disease control. In addition, evaluating changes in the peripheral T-cell repertoire of these patients would have helped to determine any association with clinical activity. Unfortunately, immunosuppressed patients were considered to be ineligible to receive ADXS11-001 since it is a live vaccine.

In conclusion, ADXS11-001 was safe and well-tolerated. Although the primary outcome was not reached, ADXS11-001 may benefit from further evaluation in combination for enhanced efficacy of therapy.

## MATERIALS AND METHODS

### Participants

Eligible patients were at least 18 years of age and had confirmed histopathologic diagnosis of squamous cell cancer of the anorectal canal that was not amenable to curative surgery. Patients must have: measurable disease according to Response Evaluation Criteria in Solid Tumors, Version 1.1 (RECIST v1.1) [38]; Eastern Cooperative Oncology Group performance status of 0 or 1; adequate hematologic, hepatic and renal function; and at least one prior line of therapy for metastatic/unresectable disease. Patients were considered ineligible if they had: central nervous system metastases and/or leptomeningeal disease; a history of listeriosis or prior ADXS11-001 therapy; HIV-positive; and a history of autoimmune disease requiring steroids or immunosuppressive agents. Patients were ineligible if they had: undergone major surgery within 6 weeks; received systemic steroid therapy and/or immunosuppressive therapy for less than 7 days or a live vaccine within 30 days. Patients with a history of a second malignancy (excluding basal cell or squamous cell carcinoma of the skin, or ductal carcinoma *in situ* of the breast) within 2 years were also ineligible. Implanted medical devices (e.g., orthopedic metal based plates) were not allowed due to presumed risk for *Listeria* colonization. 

### Study design

This was a Simon two-stage, phase II, open-label, multicenter study (NCT02399813). The overall goals of the study were to evaluate the efficacy and safety of ADXS11-001 in patients with persistent/recurrent, loco-regional or metastatic SCCA not amenable to curative surgery.

The study was conducted at 8 centers in the United States. The study was compliant with International Conference on Harmonization Good Clinical Practice guidelines for conducting, recording and reporting clinical studies [39]. The informed consent form, protocol and amendments were submitted and approved by the institutional review board or independent ethics committee for each respective investigative site.

#### Stage 1

During Stage 1, eligible patients received intravenous doses of ADXS11-001 (Advaxis, Inc., Princeton, NJ, USA) at a dose of 1 × 10^9^ colony-forming units (CFU) every 3 weeks (i.e., day 1, 22, and 43) of a 9-week treatment cycle. Treatment continued for up to 2 years, until documented disease progression or intolerable side effects. Response was evaluated per RECIST v1.1 [38] and immune-related RECIST (irRECIST) [40] at baseline and every 9 weeks thereafter, based on computed tomography and/or magnetic resonance imaging results. To assess safety, patients were monitored for adverse events as per the National Cancer Institute Common Toxicity Terminology Criteria for Adverse Events (NCI CTCAE v4.03) [41].

The study protocol mandated a total enrollment of 31 evaluable patients into Stage 1, where evaluability was defined as having at least 1 post-baseline response assessment. After 31 evaluable patients had been enrolled, further accrual was to be temporarily halted to complete an interim efficacy analysis.

#### Stage 2

If the efficacy results from Stage 1 indicated a response rate greater than or equal to 10% (by either RECIST v1.1 or irRECIST) or a 6-month PFS rate greater than or equal to 20% was observed, an additional 24 patients were to be enrolled for a total of 55 patients.

### Treatment

At least 30 minutes prior to each ADXS11-001 infusion, patients completed a prophylactic regimen to mitigate and manage potential cytokine release symptoms consistent with the mechanism of action of ADXS11-001. The regimen consisted of nonsteroidal anti-inflammatory drugs (NSAIDs), antihistamines, anti-emetics, and H2 receptor antagonists. Additional doses of NSAIDs or antiemetics were provided on days 1–2 following infusion, if needed. Furthermore, all patients received a 7-day course of antibiotic therapy (oral trimethoprim/sulfamethoxazole or ampicillin for patients with a sulfa allergy) starting 72 hours after drug administration to help ensure clearance of the attenuated *Lm* vector. In addition, a 3-year *Lm* surveillance period including a 6-month course of antibiotics following treatment discontinuation.

Patients were treated with intravenous ADXS11-001 administered over 60 minutes every 3 weeks at a dose of 1 × 10^9^ CFU for up to 2 years or until a discontinuation criterion is met. A treatment cycle is defined as 9 weeks. Tumor assessments occurred every 9 weeks.

Patients were followed for survival via a phone call every 3 months following ADXS11-001 treatment discontinuation. The end of the study was to be defined as 1 year after the last patient had been enrolled.

### Outcomes

The primary efficacy co-endpoints for the study were ORR measured by RECIST v1.1 and 6-month PFS rate. Secondary efficacy objectives were the duration of response, median PFS and overall survival (OS). The primary safety endpoint was to characterize the safety and tolerability of ADXS11-001 using NCI CTCAE v4.0 criteria.

### Statistical analyses

The study employed a 2-stage design for testing the two primary efficacy measures. The study used Simon’s method with null and alternative hypotheses H_o_: *p* ≤ .10 and H_a_: *p* > .25, whereby p represents the ORR with ADXS11-001, and determined that 31 patients needed to be enrolled in Stage 1 and an additional 24 patients be enrolled in Stage 2 (if 3 or more responses were observed in Stage 1). A 2-endpoint, 2-stage decisional framework was designed using the Two-Stage approach proposed by Benny Zee, et al. [42]: if Stage 1 demonstrates an ORR >10% or 6-month PFS > 20%, then enrollment will move to Stage 2. Using the Multinomial Two-Stage approach proposed by Benny Zee, *et al.* [42], we simulated 50,000 trials to examine the Type I and II errors under this design assumption. The following estimates, including Type I and II errors, from these simulations are: Type I error of 0.01876 with a probability of stopping early = 0.99; Type II power is 0.9277 (See Supplementary Material for more detail).

The analysis of the Stage 1 ORR and 6-month PFS data was performed on the ORR evaluable population in accordance with the protocol-defined requirements to determine if either criterion were met to proceed with Stage 2 enrollment. All primary and secondary analyses were also performed with the all enrolled patients. The degree of radiographic response was determined by the treating investigator. The primary assessment for the interim analysis was based on confirmed radiographic responses of CR and PR requiring confirmation with a subsequent diagnostic imaging study 4–8 weeks later. The 95% CIs around proportions were calculated using an exact binomial calculation. Kaplan-Meier estimates were generated in terms of the median PFS and survival probability at selected time points (e.g., 2, 4, 6 and 8 months), along with the corresponding 2-sided 95% CIs for the estimates. CIs for median OS were based upon the methods of Brookmeyer and Crowley [43]. CIs for survivorship estimates were calculated using the Greenwood formula. Disease Control Rate (DCR) was summarized descriptively, where DCR was defined as the proportion of patients with objective evidence of CR, PR or SD.

All adverse events and serious adverse events were summarized for the treatment period and for the 30-day post-treatment period. Adverse events and serious adverse events considered related to study treatment were summarized by frequency using descriptive statistics. All data summaries and listings were performed using SAS Version 9.2 under the Microsoft Windows operating system.

## SUPPLEMENTARY MATERIALS



## Abbreviations

ADXS11-001: axalimogene filolisbac
CFU: colony-forming units
CI: Confidence interval
CR: complete response
DC: disease control
DCR: disease control rate
HPV: Human papilloma virus
irRECIST: immune-related RECIST
LLO: Listeriolysin O
Lm: Listeria monocytogenes
NCI CTCAE: National Cancer Institute Common Toxicity Terminology Criteria for Adverse Events
NSAIDs: nonsteroidal anti-inflammatory drugs
ORR: overall response rate
OS: overall survival
PBMCs: peripheral blood mononuclear cells
PFS: progression-free survival
PR: partial response
RECIST: Response Evaluation Criteria in Solid Tumors
SCCA: squamous cell carcinoma of the anal canal
SD: stable disease
