# Supplementary material for: A phase II study of axalimogene filolisbac for patients with previously treated, unresectable, persistent/recurrent loco-regional or metastatic anal cancer
Source: Oncotarget. 2020 Apr 14;11(15):1334–43. doi: 10.18632/oncotarget.27536 (PMC7170499; doi:10.18632/oncotarget.27536)
Supplement: Supplementary file 1 [file oncotarget-11-1334-s001.pdf]

# A phase II study of axalimogene filolisbac for patients with previously treated, unresectable, persistent/recurrent loco-regional or metastatic anal cancer

## SUPPLEMENTARY MATERIALS

### Sample size and the design

This is a multi-center, open-label, 2 stage monotherapy study of ADXS11-001 in subjects with persistent/recurrent, loco-regional or metastatic squamous cell carcinoma of the anorectal canal (SCCA) of the anorectal canal that was previously treated in the metastatic setting.

**Primary efficacy measures are**

1. The overall response
2. Progression-Free Survival (PFS)

We employed a 2-stage design for testing these 2 primary efficacy measures. Simon's 2-stage method is not directly applicable because it is for single proportion type endpoint. However, we used Simon's method with null and alternative response rates of 10% in Stage 1 and 25% in Stage 2 to obtain the total sample sizes for both stages and use the Multinomial Two-Stage approach proposed by Benny Zee, et al. (1999) [1] to obtain the stopping probabilities.

By Simon's method, we need to enroll 31 subjects at Stage 1 and an additional 24 subjects in Stage 2 if 3 or more responses are observed in Stage 1. Based on this sample size plan, we formed the following 2 endpoints, 2-stage framework:

In Stage 1 of the study, we will enroll 31 subjects. The safety and efficacy data of the subjects will be evaluated. After 31 evaluable subjects had been enrolled, further accrual will be temporarily stopped in Stage 1 for an efficacy interim analysis evaluation. If the efficacy from Stage 1 demonstrates a response rate ≥ 10% or 6-month PFS ≥ 20%, the enrollment will re-open to Stage 2. The enrollment will be complete in Stage 2 when an additional 24 evaluable have been accrued (total from Stages 1 and 2 = 55). The Stage 1 interim results will serve as a non-binding guidance and the final decision to progress to Stage 2 will be made by the Sponsor based on the entirety of the data.

**The hypothesis testing in Stage 1 is given in the following:**

- Stage 1:
  - Null Hypothesis (H0): Response rates (r1) < 10% and 6-month PFS rate (p1) < 20%;
  - Alternative Hypothesis (Ha): Response rates (r1) ≥ 10% or 6-month PFS rate (p1) ≥ 20%
- Stage 2:
  - Null Hypothesis (H0): Response rates (r2) < 25% and 6-month PFS rate (p2) < 50%;
  - Alternative Hypothesis (Ha): Response rates (r2) ≥ 25% or 6-month PFS rate (p2) ≥ 50%

Using the Multinomial Two-Stage approach proposed by Benny Zee, et al. (1999), [1] we simulated 50,000 trials to examine the Type I and II errors under this design assumption. The following estimates, including Type I and II errors, from these simulations are summarized in the following table.

| Type I | Type II |
|---|---|
| Type I Error = 0.01876 | |
| ASN for H0 = 31.21696 | Power = 0.9277 |
| P(stop early \| H0) = 0.99096 | |

## REFERENCES


1. Zee B, Melnychuk D, Dancey J, Eisenhauer E. Multinomial phase II cancer trials incorporating response and early progression. J Biopharm Stat. 1999; 9:351–63. https://doi.org/10.1081/BIP-100101181. [PubMed]
